# Effects of spatial–temporal land cover distribution on gross primary production and net primary production in Schleswig-Holstein, northern Germany

**DOI:** 10.1186/s13021-020-00138-3

**Published:** 2020-03-19

**Authors:** Liwei Ma

**Affiliations:** 1 Department of Ecosystem Management, Institute for Natural Resource Conservation, Christian-Albrechts-Universität Zu Kiel, Olshausenstr.75, 24118 Kiel, Germany; 2 Key Laboratory of Vegetation and Environmental Change, Institute of Botany, Chinese Academy of Sciences, 20 Nanxincun, Xiangshan, Beijing, 100093 China

**Keywords:** Carbon stocks, Land cover, NPP/GPP, Calculated respiration, Hotspots and cold spots

## Abstract

**Background:**

Annual total Gross Primary Production (GPP) and Net Primary Production (NPP) and the annual total stored GPP and NPP are tightly coupled to land cover distributions because the distinct vegetation conditions of different land cover classes strongly affect GPP and NPP. Spatial and statistical analysis tools using Geographic Information Systems (GIS) were used to investigate the spatial distribution of each land cover class and the GPP and NPP based on the CORINE land cover classification in the federal state, Schleswig-Holstein, Germany for the years 2000, 2006 and 2012.

**Results:**

“Non-irrigated arable land” and “pastures” were the dominant land cover classes. Because of their large area, “non-irrigated arable land” and “pastures” had higher annual total stored GPP and NPP values than the other land cover classes. Annual total GPP and NPP hotspots were concentrated in the central-western part of Schleswig-Holstein. Cold spots were mainly located in the western and eastern Schleswig-Holstein. The distributions of the annual total GPP and NPP hotspots and cold spots were primarily determined by land cover and land cover changes among the investigated years. The average annual total NPP/GPP ratios were 0.5647, 0.5350 and 0.5573 in the years 2000, 2006 and 2012, respectively. The calculated respiration in 2006 was the highest, followed by those in 2012 and 2000.

**Conclusions:**

The land cover classes with high-ability of carbon stocks in 2000, 2006 and 2012 in Schleswig-Holstein were identified in this study. Furthermore, it is recommendable to enhance the annual total GPP and NPP and the annual total stored GPP and NPP in Schleswig-Holstein by replacing the land cover classes showing low carbon stock capabilities with the classes showing high abilities for the purpose of increasing greenhouse gas fixation.

## Highlights

- Coordination of Information on the Environment (CORINE) land cover distributions are assessed.
- Land cover classes affect annual total Gross Primary Production (GPP) and Net Primary Production (NPP).
- Locations of the hotspots and cold spots for respiration and the NPP/GPP ratio are identified.
- Land cover planning can increase GPP and NPP.

## Background

Land cover is a focal point for mapping and assessing carbon stocks [1–4] because land cover is a major driver of the distribution and function of carbon stocks, such as Gross Primary Production (GPP) and Net Primary Production (NPP). In addition, habitat fragmentation and species loss are caused by land cover and land use changes [5]. Threats to biodiversity and ecosystems are affected by land cover and land cover changes, and these threats could be minimized through better spatial planning [6]. Considering the land cover distribution is the basis for understanding the respective land cover situations and carbon stocks. The detected land cover patterns form a basic data source for interpretation and calculations to characterize the landscape potential of evaluating carbon stocks. Mapping and assessing land cover distributions are core units of the European Union (EU) biodiversity strategy [7]. Developing a primary data source for an European green infrastructure, resources to identify areas for ecosystem restoration, and a baseline for the goal of “no net loss of biodiversity and ecosystem services” are required EU-wide objectives [8, 9]. Socioeconomic factors are significant determinants of land cover distribution besides obvious affections to human society held by land cover [10, 11]. It is prominent to have a trade-off between artificial activities and influences that result from land cover distribution.

Carbon stocks are strongly affected by the environment (e.g., water, nutrients illumination) via fluctuations in the carbon concentration due to the different environmental conditions that result from distinct surface landscape conditions. GPP and NPP are the beginning of the carbon cycle in ecosystems. GPP is defined as the total carbon assimilated through photosynthesis, indicating the capacity of plants to capture carbon and energy. NPP is the net carbon stored as new biomass in ecosystems [12, 13]. As the factors that measure efficiency of storing atmospheric carbon, the ratio between GPP and NPP (NPP/GPP) and the calculated respiration, which has been defined as GPP minus NPP, are the most primary important indicators for comparing the differences between GPP and NPP [14, 15]. These indicators exhibit the abilities of ecosystems to affect carbon stocks and global climate regulation [16–18]. Calculating GPP and NPP based on land cover categories is because a land cover class can represent an ecosystem. Forest ecosystems are considered having higher annual total GPP and NPP than shrublands and croplands [19, 20], and the annual total GPP and NPP in grassland are variable because there are different grassland types [21]. In addition to the annual total GPP and NPP, the annual total stored GPP and NPP, which represent the carbon stocks in one area, vary depending on the research area [22]. In addition, the calculated respiration and the NPP/GPP ratio have been assumed to be correlated to land cover classes [23–25].

However, previous studies primarily considered the annual total GPP and NPP, the calculated respiration, the NPP/GPP ratio, and the annual total stored GPP and NPP in forests, grasslands and croplands [26–28], and some of them have been studied at the global scale [14, 29–31]. Although a large number of studies have been performed, there have been few studies on the annual total GPP and NPP, the calculated respiration, the NPP/GPP ratio, and the annual total stored GPP and NPP considering all land cover classes at regional scales. Schleswig-Holstein is an important German federal state that includes 32 out of 44 land cover classes in the CORINE land cover classification [32]. The land cover distributions and their effects on the annual total GPP and NPP, the calculated respiration, the NPP/GPP ratio, and the annual total stored GPP and NPP in different land cover classes in 2000, 2006 and 2012 can be entirely explained by the various land cover types and the continuous land cover changes among the 3 years. This study aims to provide building blocks for assessments at regional scales through a flexible method, and to ensure the EU member states support assessments in relation to the requirements for planning, agriculture, climate, water and nature policy by clarifying GPP and NPP based on the various land cover classes in Schleswig-Holstein, Germany. The objectives were to answer:

- How are different land cover classes distribute in 2000, 2006 and 2012 based on the CORINE land cover data set?
- What are the values of the annual total GPP and the annual total NPP in various land cover classes, and how do land cover classes influence the GPP and NPP distributions?
- What are the values of the respiration and the NPP/GPP ratio in 2000, 2006 and 2012 based on the classification of the CORINE land cover, and where are the hotspots and cold spots for the respiration and the NPP/GPP ratio in Schleswig-Holstein for the years 2000, 2006 and 2012?

## Methods

### Research areas

**Fig. 1:**
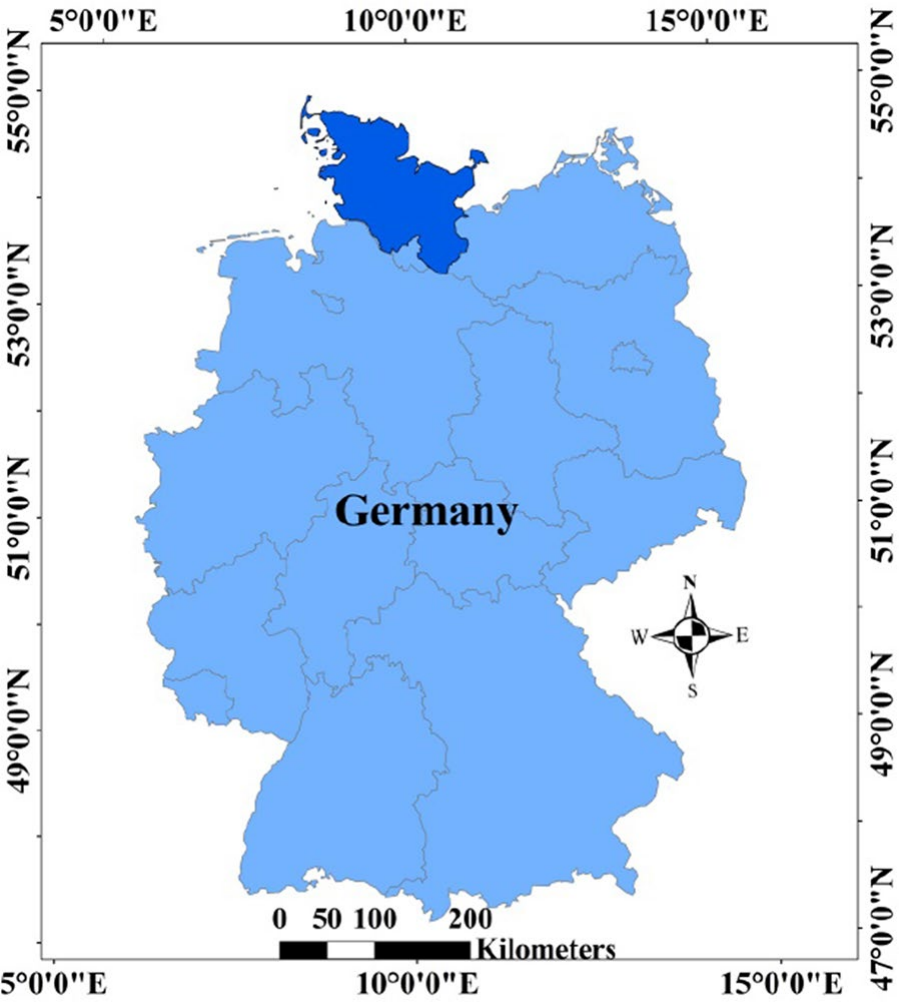
States of Germany, the state in dark blue is Schleswig-Holstein

Schleswig-Holstein (Fig. 1) is one of the 16 German federal states, locating on the northernmost national border. The state borders Denmark to the north and three German states to the south. It borders the North Sea to the west and the Baltic Sea to the east. The main landscapes are Marsch (marsh area), Geest (sandy area) and Hügelland (hill area). Alternating warmer and colder phases and the melting of glaciers in northern Germany during the Pleistocene and Holocene are the reasons for formation of landscape regions in Schleswig-Holstein [33]. There are three glaciation phases, including Saale-Elster-Weichsel, Saale and Elster moraines, in the Geest area. The Hügelland area is primarily composed of Weichsel moraines and the glacial series-Marsch, and they extended to the North Sea to form the Marsch area [33, 34].

### Data sources and methods

#### Data sources

**Fig. 2:**
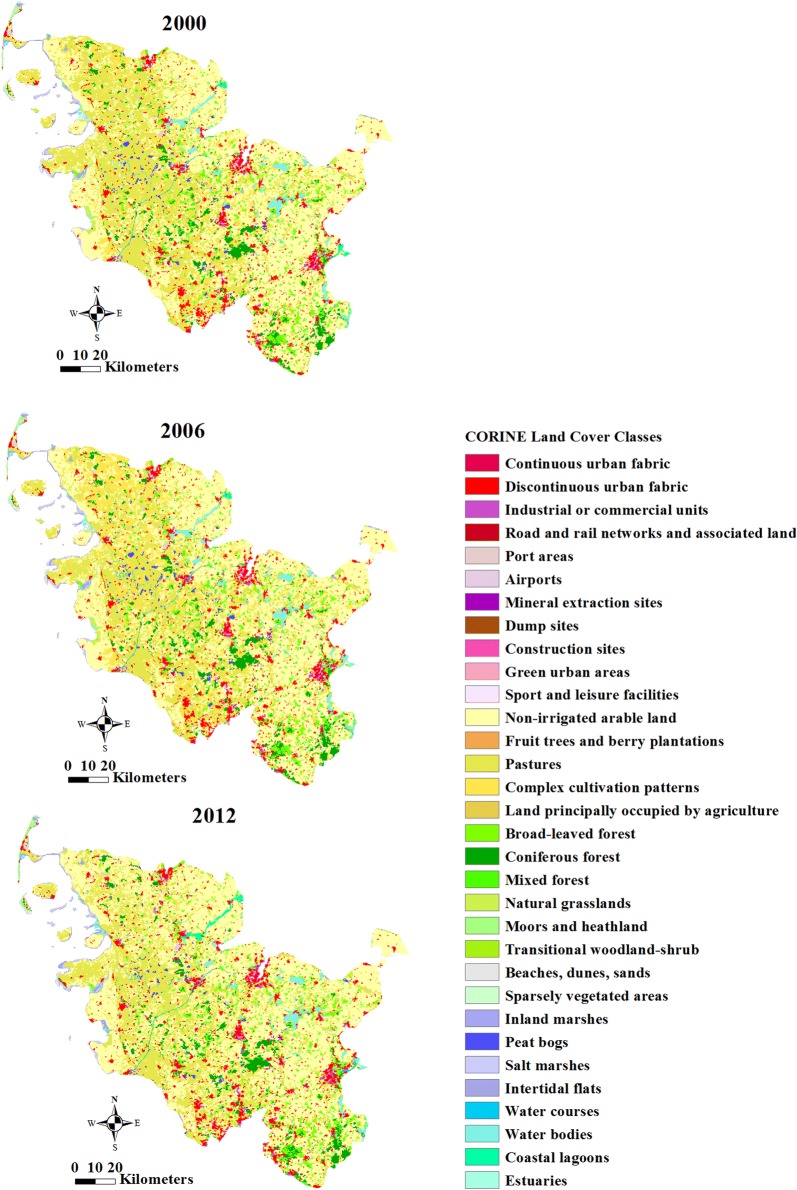
CORINE land cover maps of Schleswig-Holstein in 2000 (**a**), 2006 (**b**) and 2012 (**c**)

The CORINE land cover maps (Fig. 2) [35] are based on remote sensing data and were downloaded from the European Environmental Agency (EEA). They describe the primary land cover characteristics for whole Europe derived from long-term investigations and advanced technologies on data calibration and mapping [36, 37]. The CORINE land cover data contain an inventory of 44 (Level 3 of the CORINE Land Cover Classification) land cover classes, being available for the EU member states in 1990, 2000, 2006 and 2012 [38].

Maps of the German federal states and the borders of Schleswig-Holstein were downloaded from the Amtliches Topographisch–Kartographisches Informations-System [39]. The Moderate Resolution Imaging Spectroradiometer (MODIS) provided terrestrial satellite remote sensing images, aiming at observing parameters used for global change research that were related to ecosystem status assessment [40, 41]. GPP and NPP data were the prime components of the MOD17 products [42]. The data on the annual total GPP and the annual total NPP with 1 km × 1 km grids of Schleswig-Holstein in 2000, 2006 and 2012 were retrieved from the database of MOD17A3 at the Numerical Terradynamic Simulation Group of the University of Montana [31].

#### Methods

##### Land cover distribution

The CORINE land cover maps of Schleswig-Holstein for the years 2000, 2006 and 2012 with a resolution of 250 m were used to visualize the land cover distributions and to calculate percentage of the land cover classes using ArcGIS (the Geographic Information System). Vector data were used to calculate the area of each land cover class in Schleswig-Holstein.

##### Annual total GPP and annual total NPP classified by land cover

The MODIS GPP and NPP Project [43] were the first satellite-driven data sets to monitor vegetation productivity at a global scale. The data set supported GPP and NPP maps containing the annual total GPP and NPP at the resolution of 1 km for all continents. Therefore, it was possible to derive GPP and NPP data for Schleswig-Holstein from MODIS 17 products for carbon cycle analysis in this study. However, GPP and NPP data were unavailable in artificial areas and water bodies, because there was little vegetation cover in artificial areas and no terrestrial vegetation in water bodies [44].

The annual total GPP maps with 1 km × 1 km grids in 2000, 2006 and 2012, which were generated with ArcGIS 10.3, were used to visualize the GPP distributions in Schleswig-Holstein. The original GPP maps with 1 km × 1 km grids were resampled into maps with 250 m × 250 m grids to have the same resolution as the CORINE land cover maps. Then, the annual total GPP was computed based on the land cover classes with ArcGIS 10.3 by combining the annual total GPP maps and the CORINE land cover maps. Afterwards, for the target of calculating the annual total stored GPP and NPP (Mg C year^−1^), which were defined as the GPP stock in each land cover, the annual total stored GPP in a certain land cover (CLC area (ha)) were calculated by Eq. (1):

1 $$the\;annual\;total\; stored \;GPP=the\; annual \;total \;GPP\times CLC\;area$$

Correlations among the annual total GPP, the land cover area and the annual total stored GPP based on the land cover classes were calculated using the R software [45].

The same methods were used to map, calculate and analyze the annual total NPP and the annual total stored NPP.

##### Ratio between NPP and GPP and calculated vegetative respiration

The annual total GPP and the annual total NPP maps were produced based on the CORINE land cover classes for calculating the ratio between the NPP and GPP of each land cover class.

The vegetation respiration was derived from the difference between the annual total GPP and the annual total NPP based on the definition of the vegetation respiration [22, 31]. The vegetation respiration based on the CORINE land cover classes was evaluated by deducting the annual total NPP from the annual total GPP in ArcGIS 10.3.

##### Hotspots and cold spots of annual total GPP and annual total NPP

Hotspots are statistically significant spatial clusters of high values, and cold spots are spatial clusters of low values [46]. They were used here to identify the locations of significant hotspots and cold spots of the annual total GPP and the annual total NPP in 2000, 2006 and 2012. The data were derived from the raster data sets of MODIS annual total GPP and MODIS annual total NPP. The raster patches were converted into polygons with the raster to polygon tool in ArcGIS 10.3. Afterwards, the hot spot analysis (Getis-Ord Gi*) tool was used to identify the hotspots and cold spots of the annual total GPP and the annual total NPP. The p-values, classified with typical probabilities of 0.01, 0.05 and 0.1 measure, and the Z-scores, with 90% (b < − 1.65 or >  + 1.65), 95% (b < − 1.96 or >  + 1.96) and 99% (b < − 2.58 or >  + 2.58) confidence levels, were simply standard deviations [47]. The p-values and Z-scores indicated whether spatially clustered areas with either high or low values were more pronounced than one would expect in a random distribution of those same values.

## Results

### Land cover distributions in 2000, 2006 and 2012

The area of the different land cover and their percentages are shown in Table 1. Differences appeared in the absolute area and their relative share (percentage) in the 3 years. “Non-irrigated arable land”, “pastures” and “complex cultivation patterns” were clearly dominant land cover classes in 2000 and 2006. “Non-irrigated arable land”, “pastures” and “discontinuous urban fabric” dominated larger area than the other land cover classes in 2012, according the CORINE land cover maps. The area of “non-irrigated arable land” decreased from 666,449 ha in 2000 to 666,186 ha in 2006 and then increased to 746,016 ha in 2012. During the same period, the area of “pastures” changed from 452,238 to 367,189 ha and then to 441,388 ha from 2000 until 2006 and then to 2012. The percentage of the different land cover types had similar trends as the land cover area. The percentages of the area in “non-irrigated arable land” and in “pastures” decreased from 1990 to 2006, and then increased from 2006 to 2012. “Fruit trees and berry plantations”, “road and rail networks and associated land” and “construction sites” covered the smallest area in Schleswig-Holstein in 2000 and 2006. So did “sparsely vegetated areas”, “construction sites” and “fruit trees and berry plantations” in 2012. “Non-irrigated arable land” and “pastures” were the dominating land cover classes for the years 2000, 2006 sparsely vegetated 1 year to the other.

**Table 1:** Area and the percentage of land cover classes of Schleswig-Holstein in 2000, 2006 and 2012

Land cover classes (CORINE Level 1)	Land cover classes (CORINE Level 3)	2000		2006		2012	
		Area (ha)	Percentage (%)	Area (ha)	Percentage (%)	Area (ha)	Percentage (%)
Artificial surfaces	Continuous urban fabric	997	0.06	1030	0.07	527	0.03
	Discontinuous urban fabric	84,572	5.40	88,601	5.66	100,177	6.40
	Industrial or commercial units	7487	0.48	9076	0.58	14,877	0.95
	Road and rail networks and associated land	338	0.02	486	0.03	472	0.03
	Port areas	926	0.06	965	0.06	489	0.03
	Airports	2446	0.16	2438	0.16	2465	0.16
	Mineral extraction sites	2814	0.18	3504	0.22	2987	0.19
	Dump sites	719	0.05	807	0.05	380	0.02
	Construction sites	493	0.03	308	0.02	56	0.00
	Green urban areas	1148	0.07	1148	0.07	2063	0.13
	Sport and leisure facilities	5827	0.37	7100	0.45	9708	0.62
Agricultural areas	Non-irrigated arable land	666,449	42.59	666,186	42.57	746,015	47.67
	Fruit trees and berry plantations	259	0.02	293	0.02	244	0.02
	Pastures	452,238	28.90	367,189	23.46	441,388	28.21
	Complex cultivation patterns	93,850	6.00	164,279	10.50	743	0.05
	Land principally occupied by agriculture	24,262	1.55	27,661	1.77	6488	0.41
Forest and semi-natural areas	Broad-leaved forest	60,288	3.85	62,450	3.99	81,247	5.19
	Coniferous forest	51,808	3.31	51,759	3.31	53,196	3.40
	Mixed forest	22,005	1.41	22,863	1.46	14,302	0.91
	Natural grasslands	9400	0.60	9129	0.58	9519	0.61
	Moors and heathland	3056	0.20	3015	0.19	4477	0.29
	Transitional woodland-shrub	2132	0.14	2206	0.14	3934	0.25
	Beaches, dunes, sands	4653	0.30	4433	0.28	2032	0.13
	Sparsely vegetated areas	1321	0.08	1321	0.08	29	0.00
Wetlands	Inland marshes	4307	0.28	4480	0.29	4729	0.30
	Peat bogs	8946	0.57	9484	0.61	6623	0.42
	Salt marshes	8476	0.54	8735	0.56	11,755	0.75
	Intertidal flats	7977	0.51	8130	0.52	7485	0.48
Water bodies	Water courses	3241	0.21	3606	0.23	4308	0.28
	Water bodies	27,950	1.79	29,781	1.90	25,556	1.63
	Coastal lagoons	2817	0.18	1117	0.07	5340	0.34
	Estuaries	1716	0.11	1344	0.09	1313	0.08

### GPP distributions in Schleswig-Holstein in 2000, 2006 and 2012

**Fig. 3:**
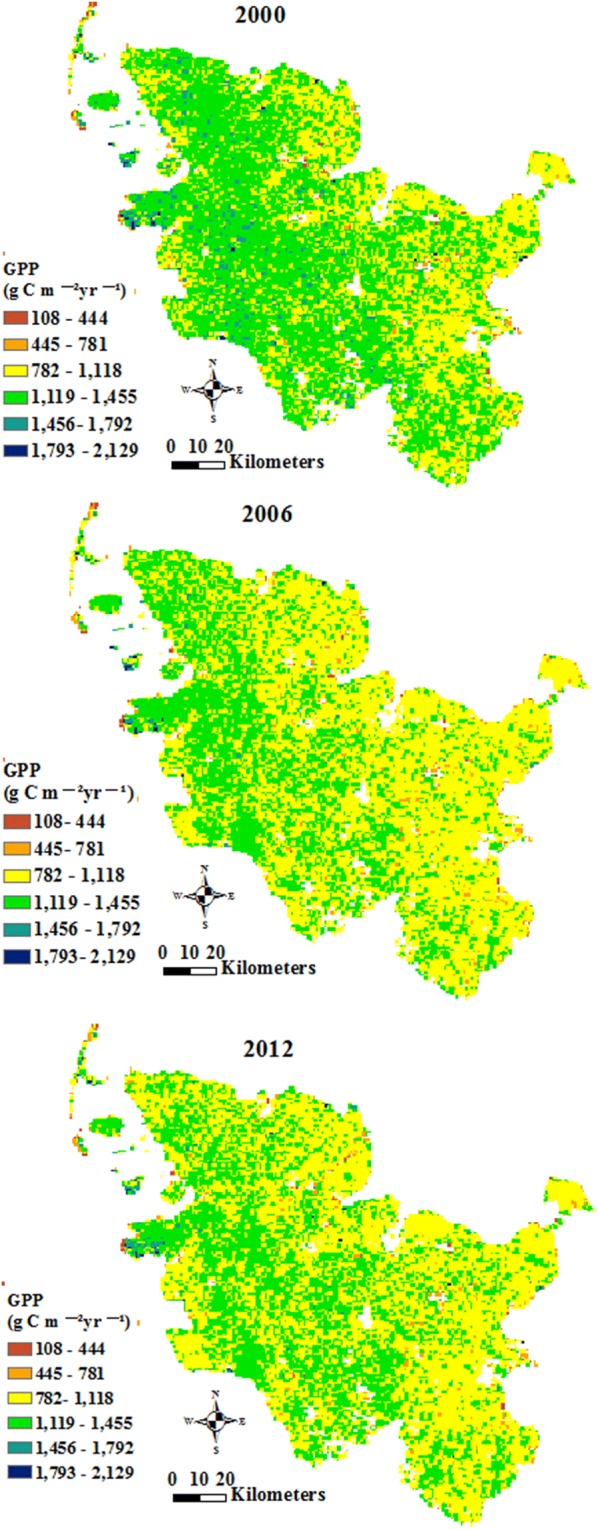
Annual total Gross Primary Production (GPP) of Schleswig-Holstein in 2000 (**a**), 2006 (**b**) and 2012 (**c**)

The annual total GPP of Schleswig-Holstein for the years 2000, 2006 and 2012 is presented in six classes in Fig. 3. The annual total GPP was the highest in 2000 and the lowest in 2006 among the 3 years, fluctuating in different regions in 2000, 2006 and 2012. The pixels with high annual total GPP were mainly in “pastures” and “coniferous forest”. The low annual total GPP appeared in “beaches, dunes and sands” and “intertidal flats”.

#### GPP distribution based on land cover classes

**Fig. 4:**
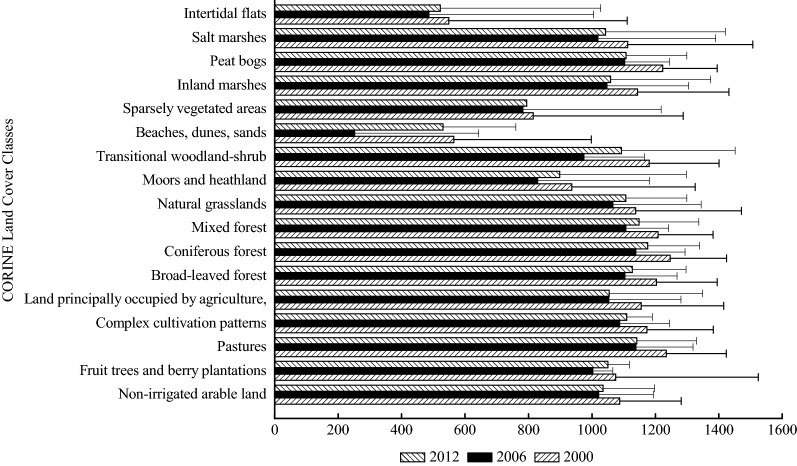
Annual total GPP (g C m^−2^ year^−1^) based on land cover classes of Schleswig-Holstein in 2000, 2006 and 2012

**Fig. 5:**
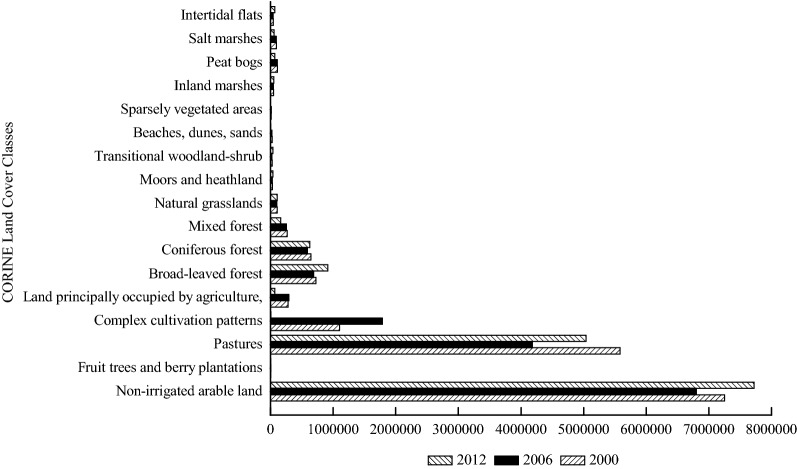
Annual total stored GPP (Mg C year^−1^) based on land cover classes of Schleswig-Holstein in 2000, 2006 and 2012

The annual total GPP presents the carbon stored with a certain spatial and temporal unit (g C m^−2^ year^−1^), while the annual total stored GPP shows the carbon stored with a certain temporal unit (Mg C year^−1^), reflecting in the spatial distribution. The annual total GPP and the annual total stored GPP in Schleswig-Holstein for the years 2000, 2006 and 2012 are shown in Figs. 4 and 5. The land cover class that had the largest annual total GPP was “coniferous forest” in 2000, followed by “pastures”, “peat bogs’, “mixed forest” and “broad-leaved forest”. The land cover classes that had the largest annual total GPP in 2006 were the same as the land cover classes in 2000. However, the value of the annual total GPP of the land cover classes in 2006 was less than the value of the annual total GPP in 2000. “Coniferous forest”, “mixed forest”, “pastures”, “broad-leaved forest” and “complex cultivation patterns” constituted the land cover classes with the largest annual total GPP in 2012.

The annual total stored GPP in 2000 was higher than those in 2006 and 2012. “Non-irrigated arable land”, “pastures”, “complex cultivation patterns”, and “broad-leaved forest” had the highest annual total GPP among the 17 land cover classes in 2000 and 2006. “Coniferous forest” replaced “complex cultivation patterns” as one of the top four land cover classes containing large amount of the annual total stored GPP in 2012. The annual total GPP and the annual total stored GPP were distinct from one land cover class to another, and either the annual total GPP or the annual total stored GPP in different years was various.

#### Correlations among annual total GPP, land cover area and annual total stored GPP

Correlation analysis among the annual total stored GPP, the annual total GPP and the land cover area showed that the annual total stored GPP was significantly affected by the other two factors. The strong effect of the land cover area indicated that the land cover area had the most important influence on the annual total stored GPP, and land cover had a considerably strong influence on the annual total GPP (Table 2).

**Table 2:** Spearman correlations among annual total GPP (g C m^−2^ year^−1^), land cover area (ha) and annual total stored GPP based on land cover classes (Mg C year^−1^)

	GPP	Land cover area	Stored GPP based on land cover
GPP	1.00	0.24*	0.26*
Land cover area		1.00	1.00**
Stored GPP based on land cover			1.00

^*^Correlation is significant at the 0.05 level

^**^Correlation is significant at the 0.01 level

### NPP of Schleswig-Holstein in 2000, 2006 and 2012

**Fig. 6:**
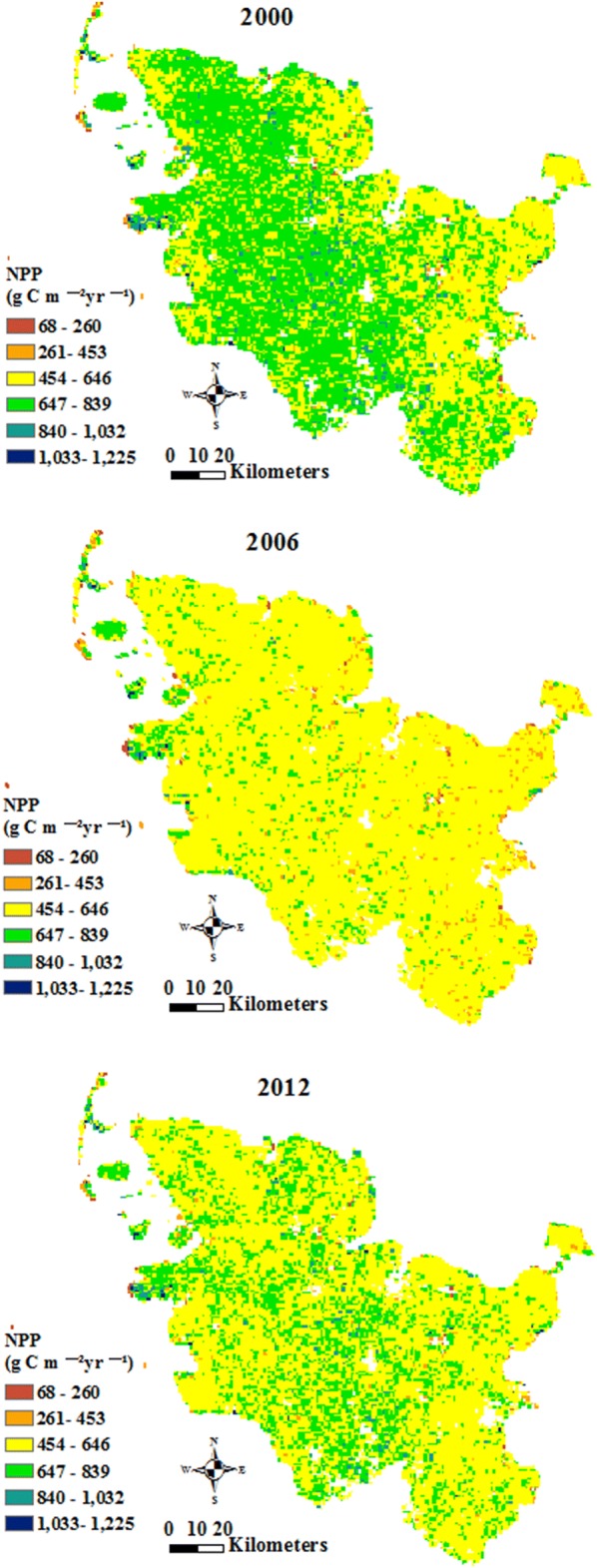
Annual total Net Primary Production (NPP) of Schleswig-Holstein in 2000 (**a**), 2006 (**b**) and 2012 (**c**)

The annual total NPP represents the NPP stored in biomass with a special unit in 1 year, and the annual total stored NPP is the amount of NPP in one land cover class. Figure 6 presents the annual total NPP maps of Schleswig-Holstein in 2000, 2006 and 2012. The annual total NPP in 2000 was larger than that in 2012 and 2006, and was the lowest in 2006.

#### NPP distributions based on land cover classes

**Fig. 7:**
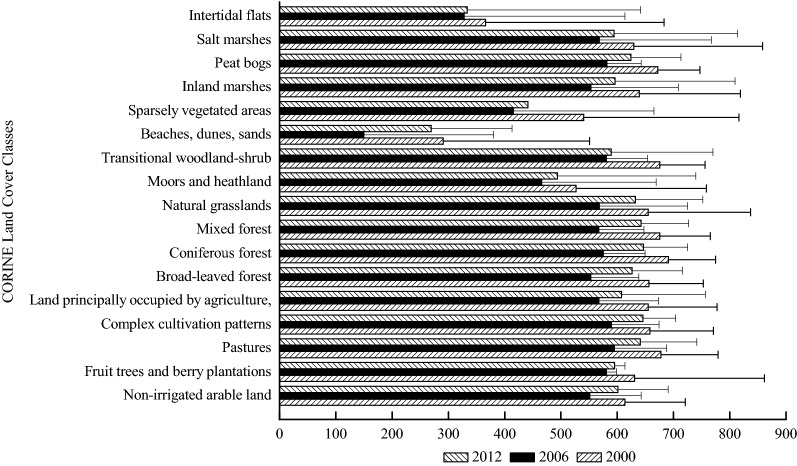
Annual total NPP (g C m^−2^ year^−1^) based on land cover classes of Schleswig-Holstein in 2000, 2006 and 2012

**Fig. 8:**
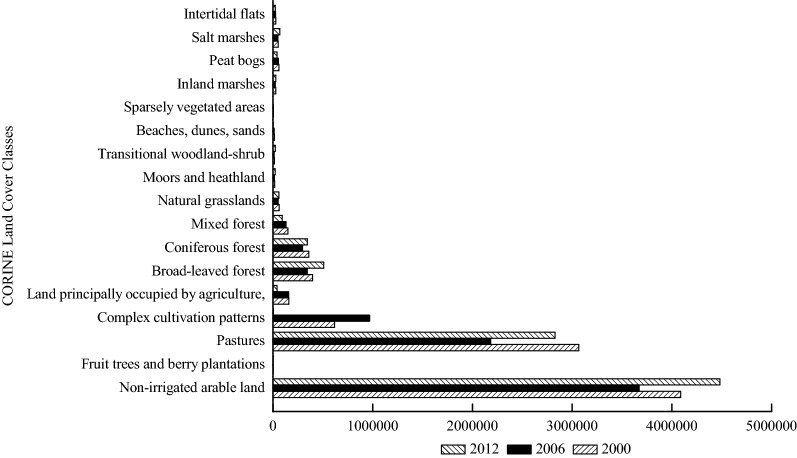
Annual total stored NPP (Mg C year^−1^) based on land cover classes of Schleswig-Holstein in 2000, 2006 and 2012

The carbon stored in vegetation with a certain spatial and temporal unit is denoted by the annual total NPP. Figures 7 and 8 present the annual total NPP and the annual total stored NPP based on land cover classes for the years 2000, 2006 and 2012. “Pastures”, “mixed forest”, “transitional woodland-shrub” and “peat bogs” had the largest annual total NPP in 2000. “Pastures”, “complex cultivation patterns”, “transitional woodland-shrub”, “peat bogs” and “fruit trees and berry plantations” and “coniferous forest” produced the highest annual total NPP in 2006. “Coniferous forest”, “complex cultivation patterns”, “mixed forest”, “pastures” and “natural grasslands” had the largest annual total NPP in 2012.

The annual total stored NPP had the highest and the lowest values in 2000 and 2006, respectively. “Non-irrigated arable land”, “pastures”, “complex cultivation patterns”, and “broad-leaved forest” had the highest annual total NPP among the land cover types for the years 2000 and 2006. “Coniferous forest” took the position of “complex cultivation patterns” as one of the top four land cover classes that contained large amount of the annual stored NPP in 2012.

#### Correlations among annual total NPP, land cover area and annual total stored NPP

The annual total NPP and the land cover area, which might influence the annual total stored NPP were analyzed using correlation analysis. Table 3 presents that the annual total NPP and the land cover area significantly affected the annual total stored NPP. The land cover area had the most important effect on the annual total stored NPP as indicated by the very high coefficient value.

**Table 3:** Correlations among annual total NPP (g C m^−2^ year^−1^), land cover area (ha) and annual total stored NPP spearman correlations based on land cover classes (Mg C year^−1^)

	NPP	Land cover area	Stored NPP based on land cover
NPP	1.00	0.24*	0.25*
Land cover area		1.00	1.00**
Stored NPP based on land cover			1.00

^*^Correlation is significant at the 0.05 level

^**^Correlation is significant at the 0.01 level

### Annual total NPP/GPP, and calculated respiration based on land cover classes

**Fig. 9:**
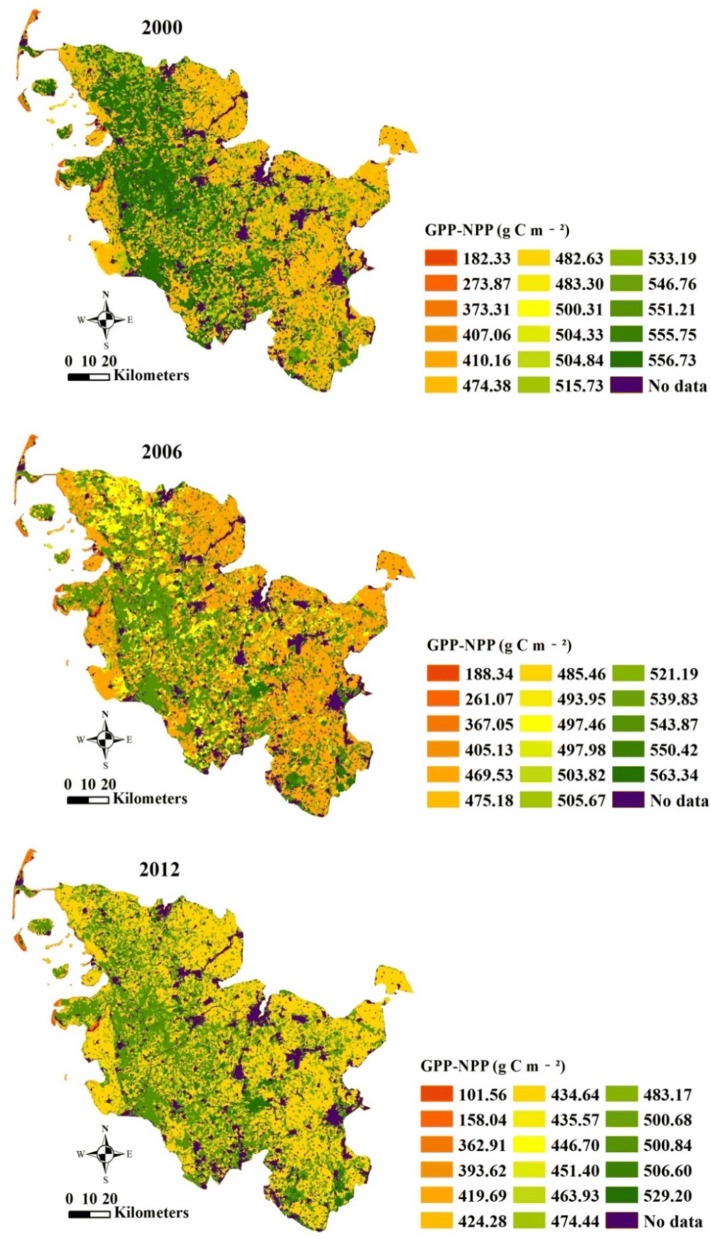
Maps of calculated respiration based on land cover classes of Schleswig-Holstein in 2000 (**a**), 2006 (**b**) and 2012 (**c**)

The calculated respiration by green plants per unit of time and space was estimated as the difference between the annual total GPP and NPP. The respiration maps based on CORINE land cover classes in Schleswig-Holstein for the years 2000, 2006 and 2012 are shown in Fig. 9 and Table 4. The respiration in 2006 was the highest, and 2012 had the lowest respiration among the 3 years. The lowest calculated respiration appeared in “intertidal flats” and “beaches, dunes, sands”, shown in red in Fig. 9. In contrast, the ratio between respiration and GPP in 2006 was lower than those in 2000 and 2012. These ratios in the land cover class of “intertidal flats” were 33.25%, 36.09% and 32.52% for the years 2000, 2006 and 2012, respectively. “Intertidal flats” had the lowest ratio between respiration and GPP. “Beaches, dunes, sands” (48.50% in 2000 and 49.23% in 2006) and “sparsely vegetated areas” (50.62%) had the greatest ratios for the years 2000, 2006 and 2012. These results indicated that more energy was been fixed by autotrophs in “intertidal flats” than in “beaches, dunes, sands”, “broad-leaved forest” or “sparsely vegetated areas”.

**Table 4:** Calculated respiration and ratios between annual total NPP and GPP based on land cover classes of Schleswig-Holstein in 2000, 2006 and 2012

Land cover classes	Calculated respiration between GPP and NPP (GPP–NPP) (g C m^−2^ year^−1^)			Ratios between GPP and NPP (NPP/GPP)		
	2000	2006	2012	2000	2006	2012
Non-irrigated arable land	474.38	469.53	434.64	0.5639	0.5400	0.5802
Fruit trees and berry plantations	407.06	493.95	419.69	0.5938	0.5403	0.6004
Pastures	556.73	543.87	500.84	0.5490	0.5222	0.5613
Complex cultivation patterns	515.73	497.46	463.93	0.5606	0.5424	0.5818
Land principally occupied by agriculture	500.31	485.46	446.70	0.5669	0.5388	0.5763
Broad-leaved forest	546.76	550.42	500.68	0.5454	0.5011	0.5556
Coniferous forest	555.75	563.34	529.20	0.5541	0.5050	0.5498
Mixed forest	533.19	539.83	506.60	0.5588	0.5123	0.5590
Natural grasslands	482.63	497.98	474.44	0.5757	0.5327	0.5713
Moors and heathland	410.16	405.13	362.91	0.5621	0.5492	0.5620
Transitional woodland-shrub	504.84	503.82	393.62	0.5722	0.5390	0.5959
Beaches, dunes, sands	273.87	132.92	101.56	0.5149	0.5077	0.5957
Sparsely vegetated areas	373.31	367.05	435.57	0.5416	0.5308	0.4938
Inland marshes	504.33	505.67	451.40	0.5590	0.5224	0.5689
Peat bogs	551.21	521.19	483.17	0.5494	0.5274	0.5636
Salt marshes	483.30	475.18	424.28	0.5656	0.5444	0.5834
Intertidal flats	182.33	188.34	158.04	0.6675	0.6391	0.6747

**Fig. 10:**
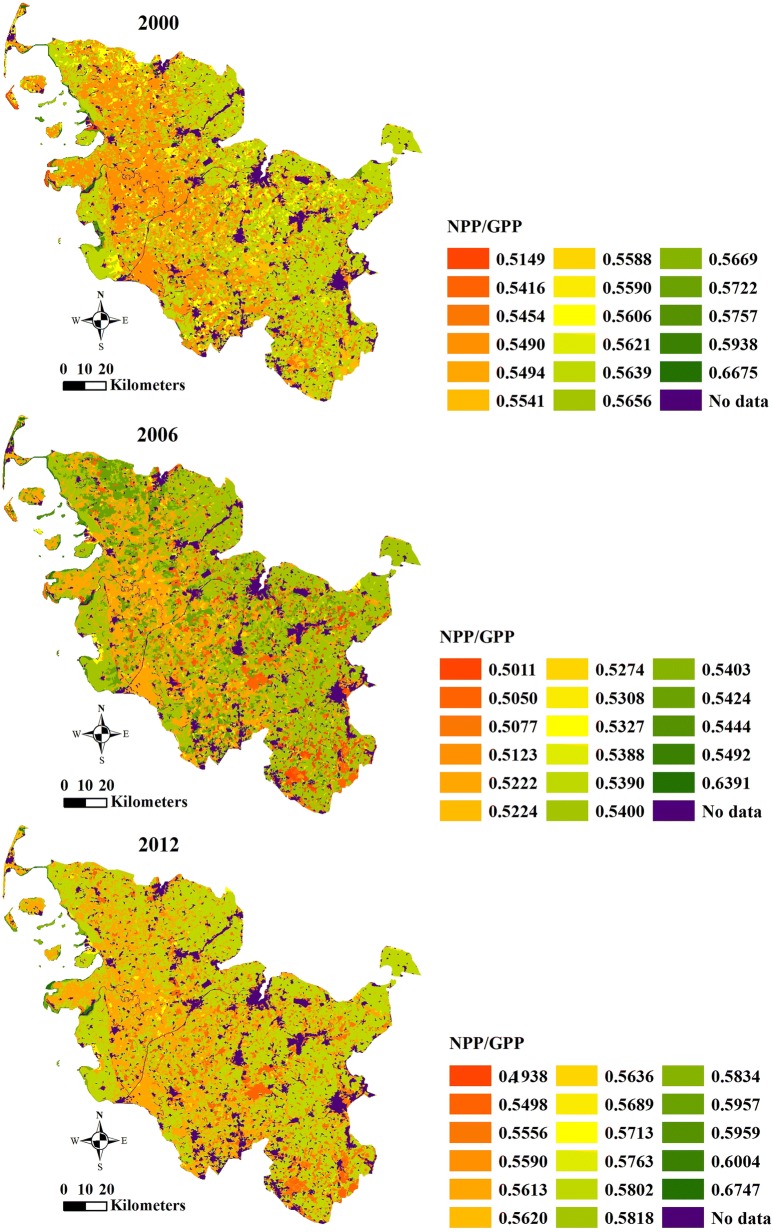
Maps of ratio between annual total NPP and GPP based on land cover classes of Schleswig-Holstein in 2000 (**a**), 2006 (**b**) and 2012 (**c**)

The annual total NPP/GPP based on the land cover classes for the years 2000, 2006 and 2012 showed fluctuations (Fig. 10 and Table 4). The average ratios of the annual total NPP/GPP were 0.5647, 0.5350 and 0.5573 in 2000, 2006 and 2012, respectively. The range between the minimum and the maximum of the annual total NPP/GPP in 2006 was larger than that in 2000 but smaller than that in 2012. The annual total NPP/GPP was different from one land cover class to another in Schleswig-Holstein for the years 2000, 2006 and 2012. “Intertidal flats” had the maximum annual total NPP/GPP (0.6675 in 2000, 0.6391 in 2006 and 0.6747 in 2012). “Beaches, dunes, sands” (in 2000 and 2006) and “sparsely vegetated areas” (in 2012) were the land cover classes with the minimum ratios, shown in red in Fig. 10. The land cover classes with the minimum and maximum values of the annual total NPP/GPP were the same as the land cover classes those had the highest and lowest ratios between respiration and GPP.

### Identifying hotspots and cold spots for annual total GPP and annual total NPP of Schleswig-Holstein

**Fig. 11:**
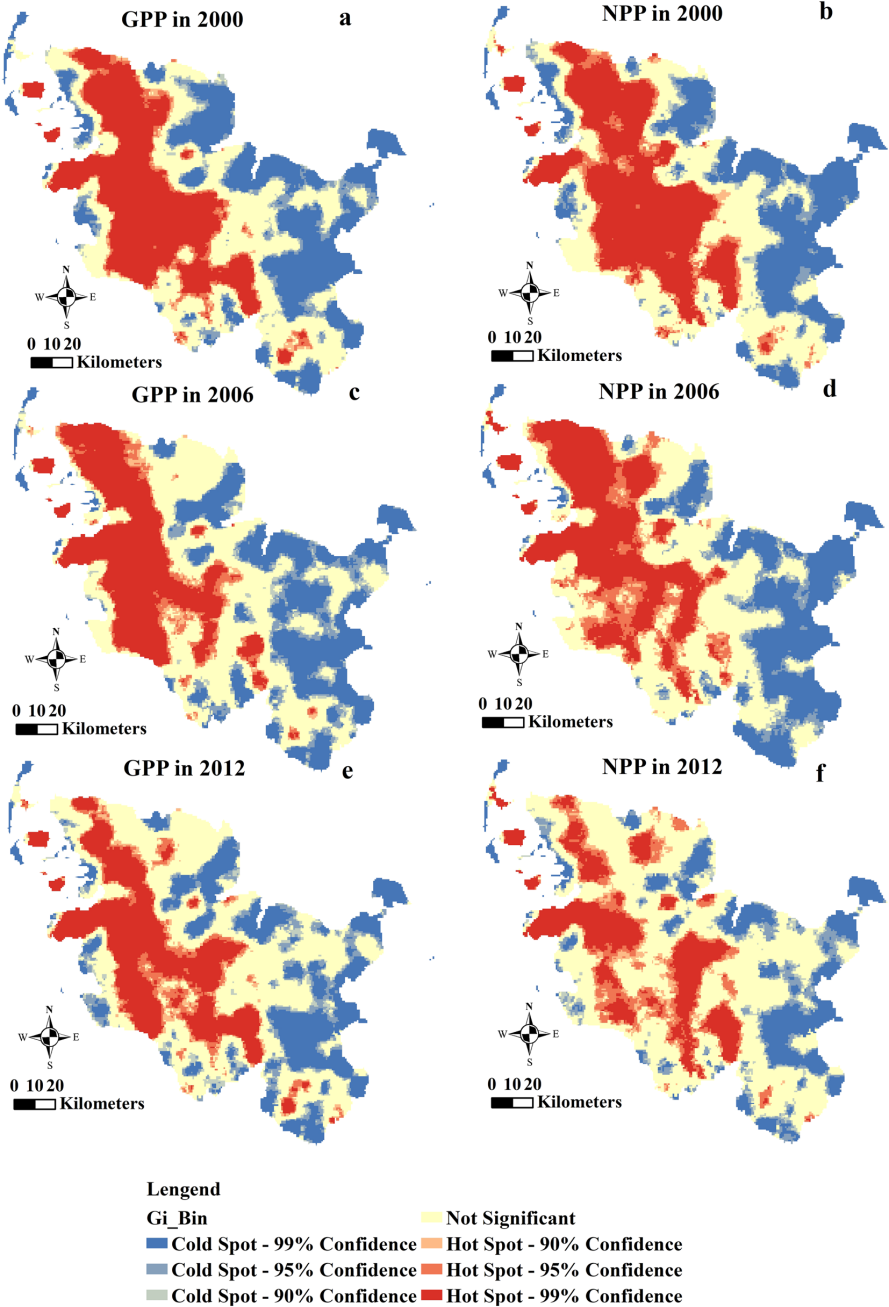
Spatial distributions of hotspots and cold spots for annual total GPP (**a**, **c** and **e**) and NPP (**b**, **d** and **f**) of Schleswig-Holstein in 2000, 2006 and 2012

Estimating the spatial distributions of the hotspots and cold spots for the annual total GPP and NPP presents core distribution areas. The spatial distributions of the hotspots and cold spots for the annual total GPP and the annual total NPP in Schleswig-Holstein for the years 2000, 2006 and 2012 show core distribution areas (Fig. 11). The hotspot areas were in the central-western to the central-southern areas of Schleswig-Holstein in 2000, 2006 and 2012, forming an adjacent significant hotspot area with high annual total GPP. The cold spotareas primarily occupied the edges of the western and eastern parts of the federal state. The areas of the hotspots and the cold spots fluctuated during the 3 years (Table 5). Approximately 36.12%, 32.69% and 32.38% of the state’ areas were distributed in the identified hotspot areas for the years 2000, 2006 and 2012, respectively. Meanwhile, the cold spots accounted for 33.72%, 33.84% and 30.73% of the total areas of the state in the 3 years, respectively. The percentage of non-significant areas increased from 30.16% in 2000 to 33.47% in 2006 and then reached 36.89% in 2012. The decrease of hotspots areas and extension of the areas of non-significant results indicated a fluctuation in the cold spot areas during the study periods. The distributions of the hotspots and the cold spot areas of the annual total NPP showed similar trends as the distribution of the annual total GPP, covering areas from the central-western to the central-southern areas of the state in 2000, 2006 and 2012. Furthermore, the distribution of hotspot areas in 2012 were much more fragmented than the distributions in 2000 and in 2006, besides that the areas occupied by the hotspots of the annual total NPP declined during the study period. Contrarily, the growth of the non-significant areas proceeded rapidly, increasing from 28.93% of the total area of the state in 2000 to 44.94% in 2012. The percentage of the cold spot areas (around 31.10%) was similar in 2000 and 2006, and then decreased to 24.73% in 2012. However, the decline in the areas of the hotspot and cold spot areas was much more significant for the annual total NPP than the decrease of the areas of the annual total GPP.

**Table 5:** Summary of the analysis for hotspot and cold spot areas of annual total GPP and NPP

	Year	Area of hotspot and cold spot (ha)						
		Cold spot-99% confidence	Cold spot-95% confidence	Cold spot-90% confidence	Not significant	Hotspot-90% confidence	Hotspot-95% confidence	Hotspot-99% confidence
GPP	2000	382,049	94,615	51,525	472,347	35,770	66,739	463,200
	2006	359,810	113,410	56,736	524,258	43,768	73,461	394,801
	2012	307,043	112,834	61,456	577,752	56,269	91,513	359,379
NPP	2000	353,639	90,586	42,932	453,082	41,829	91,933	492,245
	2006	352,170	95,339	40,060	482,294	59,269	141,421	395,691
	2012	223,674	106,650	57,035	703,831	72,384	136,637	266,034

## Discussion

The CORINE land cover maps have been widely used for assessing ecological factors and stability at the European, national, regional and local scales [14, 48–50], or for evaluating ecosystem services [32, 51]. The CORINE land cover maps of Germany were mapped with a specific national approach, using ATKIS [52]. Therefore, CORINE land cover maps are well adapted for studies for broader extents and resolutions. The findings in this study showed that “non-irrigated arable land” and “pastures” were the land cover classes that occupied the largest amount of area in Schleswig-Holstein for the years 2000, 2006 and 2012 (Table 1). The outcomes agree with results about land cover and land use distributions in the Bornhöved Lakes, Northern Germany, estimated with multiple data sources [53]. This distribution trend resulted from the significant dominance of agricultural areas in Schleswig-Holstein due to social-economic planning by the federal government [54]. The wide distributions of “non-irrigated arable land” and “pastures” were strengthened by the European Commission Policies, for instance by the Common Agricultural Policy (CAP) [26]. These results are in agreement with Rounsevell et al. [55], who found that over 50% of the surface area of the European Union was covered by agriculture.

Land cover distributions are driven by complex environmental, social and economic conditions that are focal points for sustainable land cover plans [56, 57]. The distributions frequently change due to human activities that have been increasingly influenced by political plans during the last few centuries, for instance urbanization or intensively using agricultural land [37, 58]. Land cover classes in Schleswig-Holstein are often initiated by the cultivation of silage maize for biogas plants that have been a widely discussed political issue in Germany. As a renewable energy source (RES) system in Germany, the system was established in 1980 and became a consensual aim in 2010 because of the continuously growing number of beneficiaries [59]. An enormous number of biogasplants grown monoculture for electricity production were produced from the 1980s to 2000s, enhancing the annual GPP and NPP stocks. However, the wide production of biogas plants induces to erosion, eutrophication, soil carbon loss and monotonous landscape [60]. Take cultivating silage maize for example, it may be an important factor which provides the highest overall impacts on land cover and land cover changes since the 1980s because of the policy [61]. Following the transitional energy regime, the number of biogas plants in Germany significantly grew 57.6 times from 1992 until 2013 [62]. As the critically cultivated area of biogas plants, pastures and maize has expended rapidly in recent decades [63, 64]. Schleswig-Holstein has widely covered by “pastures”, “non-irrigated arable land” or “complex cultivation patterns”, because of a prime step for developing RES.

The land cover classes in Schleswig-Holstein which had the largest annual total GPP and NPP (Figs. 4 and 7) were “coniferous forest”, “mixed forest”, “broad-leaved forest”, and “pastures”. The annual total NPP in this study had similar results as previous studies about NPP (456.8 g C m^−2^ year^−1^ NPP in needle-leaf forest, 613.1 g C m^−2^ year^−1^ NPP in broad-leaf forest, 559.5 g C m^−2^ year^−1^ g C m^−2^ year^−1^ NPP in mixed forest and 122.6 in grass g C m^−2^ year^−1^ NPP in grass land) in Chinese terrestrial ecosystems, representing that the annual total NPP in forests was higher than in grassland [22, 26]. The values of the annual total NPP in the three types of Chinese forests and one grassland were lower than the annual total NPP of the three land cover types in Schleswig-Holstein because the geophysical and geochemical conditions in China were much more heterogeneous than those in Schleswig-Holsten. The heterogeneous conditions may lead to negative influences on the annual total GPP and NPP through affecting vegetation growth [65]. In addition to the distinctions of the annual total GPP and NPP owing to the locaton of the study area, nutrients availability which were imported during the process of fertilization, led to “pastures” showing a similar performance trend as forest (Table 1) [6, 58]. However, the annual total GPP and the annual total NPP in the other land cover classes were different from the annual total GPP and the annual total NPP in pastures and forests. The reasons for the differences among the 17 land cover classes are that the land cover changes may have impacts on albedo, evapotranspiration, and sources and sinks of gases which are the ingredients for biological carbon sequestration [66].

The annual total stored GPP and the annual total stored NPP (Figs. 6 and 9) in “non-irrigated arable land” and “pastures” were much higher than those in the other land cover classes although the annual total GPP and the annual total NPP in “non-irrigated arable land” and “pastures” were close to those in the other land cover classes. The distinctions of the annual total stored GPP and the annual total stored NPP among the various land cover classes illustrate that the influences from the annual total GPP and the annual total NPP on the annual total stored GPP and the annual total stored NPP were not as significant as the influences from the land cover area (Tables 2 and 3). These results suggest that the land cover distributions and changes significantly influence biological sequestration [23, 67, 68], such as GPP and NPP. The higher of the annual total stored GPP and the annual total stored NPP values imply more carbon fixation and lower CO_2_ emissions. Hence, the land cover managers of Schleswig-Holstein can increase greenhouse gas fixation (e.g. CO_2_) through afforestation and sustainable intensification of agriculture, which would lead to increases in areas of “broad-leaved forest”, “coniferous forest”, “mixed forest”, “pastures” and “non-irrigated arable land”. However, this will be a long-term process due to making agreements of property rights on land cover and land use changes by landholders and governmental managers in Germany [69]. Therefore, providing payments to landholders whose land cover and land use area would be decreased, and increasing the areas of “broad-leaved forest”, “coniferous forest”, “mixed forest”, “pastures” and “non-irrigated arable land”, might be good options [70, 71].

The annual respiration from plants was calculated as the difference between GPP and NPP [20] (Fig. 10). The calculated respiration on the land cover classes of Schleswig-Holstein ranged from approximately 182.33 g C m^−2^ year^−1^ to 556.73 g C m^−2^ year^−1^ in 2000, from 188.34 to 563.34 g C m^−2^ year^−1^ in 2006, and from 101.56 to 529.20 g C m^−2^ year^−1^ in 2012. However, the calculated respiration and GPP for different ages of forests ranged from 230 to 340 g C m^−2^ year^−1^ [15, 19]. The large differences in these calculated respiration values resulted from the multiple land cover classes in the study and the single land cover class (forest) in the study of Goulden et al. [19].

The NPP/GPP ratios, which are critical for understanding the carbon stocks of ecosystems and their responses to climate change [64, 72], were between 0.5011 and 0.6774 in Schleswig-Holstein. A study on NPP/GPP ratios at the global scale found that the ratio fluctuated around an average of 0.5, and the ratio stabilized at approximately 0.61 between 30° and 60° in the Northern Hemisphere [20]. The NPP/GPP ratios of Schleswig-Holstein for the years 2000, 2006 and 2012 matched these results, because that the land cover classes included in this study are typical land cover classes between 30° and 60° in the Northern Hemisphere.

The maps of the annual total GPP and the annual total NPP hotspots and cold spots indicated that the annual total GPP and the annual total NPP for the years 2000, 2006 and 2012 were not scattered randomly across Schleswig-Holstein but rather occurred in particular patterns (Fig. 11). The hotspots depict areas with high levels of the annual total GPP and the annual total NPP, and the cold spots present areas with low levels of the annual total GPP and the annual total NPP. However, the distributions of hotspots and cold spots are classified based on statistical values, their distributions indicate areas that have high or low value in one temporal–spatial condition [73]. The locations of the “pastures” that changed from 2000 to 2006, owing to crop rotation, matched to the hotspot loss of the annual total GPP and the annual total NPP from 2000 to 2006. The absent of “Complex cultivation patterns”, which represented by elaborate cultivation areas, resulting in the hotspots loss of the annual total GPP and the annual total NPP from 2006 to 2012 due to the sharp area decline of “complex cultivation patterns” from 2006 until 2012. Enhancing the annual total GPP and the annual total NPP in western and eastern Schleswig-Holstein may increase the annual total stored GPP and the annual total stored NPP. Expending the land cover area of “pastures” and “complex cultivation patterns” is critical to increase the annual total stored GPP and the annual total stored NPP, which are representative of the ability of the landscape for fixing CO_2_.

Uncertainties originating from various inputs, such as land cover data, the fraction of the absorbed photosynthetic active radiation or the leaf area index, meteorological data, and the algorithm itself, can influence the accuracy of the GPP and NPP products [74, 75]. Furthermore, the annual total GPP and the annual total NPP distributions based on the CORINE land cover classes in our study were derived from the global estimation system with a resolution of 1 km × 1 km. The accuracy of the annual total GPP and the annual total NPP for some land cover classes with rare area may be deduced with the downscaling calculation from the global to the regional scale [76]. Considering the uncertainties in the regional assessments in Schleswig-Holstein, the recommended approach for managing uncertainties in the assessments is to collect high-quality and complete input data for the analysis. Furthermore, improving the estimation methodology for the annual total GPP and the annual total NPP is also important for uncertainty reduction.

## Conclusion

In this study, for the years 2000, 2006 and 2012, the distributions of land cover classes based on the CORINE land cover data set, their influences on the GPP and NPP represented by the annual total GPP and the annual total NPP, the respiration and the NPP/GPP ratio based on the CORINE land cover classification, and the hotspots and cold spots of the respiration and the NPP/GPP ratios in Schleswig-Holstein were analyzed. The results presenting the land cover distributions in Schleswig-Holstein showed that “non-irrigated arable land”, “pastures” and “complex cultivation patterns” were the dominant land cover classes for the years 2000 and 2006. “Non-irrigated arable land”, “pastures” and “discontinuous urban fabric” occupied much more area than the other land cover classes in 2012. “Pastures” and “non-irrigated arable land” were the most widely distributed land cover classes in Schleswig-Holstein.

The annual total GPP and NPP, the annual total stored GPP and NPP and the hotspots and cold spots of annual total GPP and NPP indicated the capacity of the carbon stocks in Schleswig-Holstein. The hotspots and cold spots of annual total GPP and NPP formed the adjacent significant areas with high annual total GPP and NPP, located from the central-western to the central-southern areas of Schleswig-Holstein. The cold spot areas with low annual total GPP and NPP were primarily located at the western edge and in the eastern part of the state. The calculated respiration in 2006 was higher than that in 2000 and 2012, and 2000 had the lowest respiration among the 3 years. The average ratios of the annual NPP/GPP were 0.5647, 0.5350 and 0.5573 in 2000, 2006 and 2012, respectively.

The findings reveal the carbon stocks in this area, as evaluated with the annual total GPP, the annual total NPP, the calculated respiration and the NPP/GPP ratio based on the CORINE land cover classes. Higher annual total stored GPP and NPP values mean more carbon fixation, and lower CO_2_ emissions. Hence, the land cover managers of Schleswig-Holstein can increase greenhouse gas fixation (e.g. CO_2_) by increasing the areas of “broad-leaved forest”, “coniferous forest”, “mixed forest”, “pastures” and “non-irrigated arable land”.

## Data Availability

The border maps of Germany and Schleswig-Holstein used in the current study are available in the ATKIS repository, https://www.adv-online.de/Products/Geotopography/ATKIS/ (accessed 11 April 2013) [40]. CORINE land cover maps for Europe are available from https://land.copernicus.eu/pan-european/corine-land-cover (accessed 20, May 2013) [36]. Gross Primary Production and Net Primary Production data are accessed from Project (MOD17) [44]. https://files.ntsg.umt.edu/data/NTSG_Products/MOD17/ (accessed 17 August 2014).

## Abbreviations

GPP: Gross Primary Production
NPP: Net Primary Production
GIS: Geographic Information Systems
CS: Carbon stocks
EU: European Union
NPP/GPP: A ratio between GPP and NPP
EEA: European Environmental Agency
CORINE: Coordination of information on the environment
ATKIS: Amtliches Topographisch–Kartographisches Informations-System
CLC: CORINE land cover
CAP: Common agricultural policy
RES: Renewable energy sources
